# Induction of mitochondrial damage via the CREB3L1/miR-34c/COX1 axis by porcine epidemic diarrhea virus infection facilitates pathogenicity

**DOI:** 10.1128/jvi.00591-24

**Published:** 2025-03-12

**Authors:** Hangao Xie, Ting Xiong, Jinlian Guan, Yin Han, Haixia Feng, Fei Xu, Sixuan Chen, Jiahui Li, Ziwei Xie, Dingxiang Liu, Ruiai Chen

**Affiliations:** 1 College of Veterinary Medicine, South China Agricultural University 554665 , Guangzhou, China; 2 Zhaoqing Branch Centre of Guangdong Laboratory for Lingnan Modern Agricultural Science and Technology, Zhaoqing, China; 3 Integrative Microbiology Research Centre, South China Agricultural University Integrative Microbiology Research Centre 578635 , Guangzhou, China; 4 Key Laboratory of Manufacture Technology of Veterinary Bioproducts, Ministry of Agriculture and Rural Affairs 12654 , Beijing, China; 5 Guangdong Enterprise Key Laboratory of Biotechnology R&D of Veterinary Biologics, Zhaoqing, China; 6 Zhaoqing Dahuanong Biology Medicine Co. Ltd., Zhaoqing, China; Loyola University Chicago - Health Sciences Campus, Maywood, Illinois, USA

**Keywords:** porcine epidemic diarrhea virus (PEDV), CREB3L1, miR-34c, COX1, mitochondrial damage

## Abstract

Porcine epidemic diarrhea virus (PEDV) is a primary cause of viral diarrhea in neonatal piglets, leading to substantial economic losses in the swine industry globally. It primarily targets epithelial cells of the small intestine, compromising intestinal function and resulting in the death of affected animals. As mitochondria are essential for maintaining gut health, this study investigates the effects of PEDV infection on mitochondrial function in small intestinal epithelial cells and its subsequent impacts. Using small RNA sequencing, fluorescence *in situ* hybridization, dual luciferase reporter assay, gene overexpression, and silencing experiments, we investigated the mitochondrial structural and functional impairments induced by PEDV infection in jejunum epithelial cells of piglets and characterized the regulatory pattern of miRNAs in mitochondria of jejunum epithelial cells during PEDV infection. The results indicate that PEDV infection leads to the upregulation and mitochondrial localization of the nuclear-encoded microRNA, miR-34c, which in turn suppresses COX1 expression. The activation of the miR-34c/COX1 axis diminishes mitochondrial complex III, IV, and V activities, depletes ATP, lowers mitochondrial oxygen consumption, induces mitochondrial depolarization, increases the accumulation of mitochondrial reactive oxygen species (mtROS), and stimulates mitophagy. Furthermore, we confirm that CREB3L1 acts as an upstream transcription factor regulating the miR-34c/COX1 axis during PEDV infection, modulating mitochondrial damage in the epithelial cells of the jejunum. These findings demonstrate for the first time that PEDV infection activates the miR-34c/COX1 axis via the transcription factor CREB3L1 and regulates the nuclear-mitochondrial communication and mitochondrial fate, providing a new perspective on the pathogenesis of PEDV.

**IMPORTANCE:**

This study reveals the mechanism by which the porcine epidemic diarrhea virus (PEDV) disrupts mitochondrial function in piglets, enhancing viral pathogenicity. By demonstrating how PEDV infection upregulates miR-34c, leading to COX1 suppression and subsequent mitochondrial dysfunction, the research highlights a novel aspect of viral manipulation of host cellular mechanisms. These findings provide a deeper understanding of the PEDV pathogenesis and identify potential targets for therapeutic intervention, advancing efforts to mitigate the economic impact of PEDV on the swine industry.

## INTRODUCTION

Porcine epidemic diarrhea virus (PEDV) is an enteric pathogen exclusively transmitted among pigs, causing infection and mortality. The acute viral intestinal infection causes diarrhea outbreaks and mortality in neonatal piglets worldwide, with PEDV being the primary causative agent of acute infectious diarrhea in this population (1, 2). The primary mode of PEDV transmission is via the fecal-oral route, with a high concentration of virions found in the intestines of infected swine (3). The mucosal barrier, formed by the tight junctions between porcine intestinal epithelial cells and adjacent intestinal epithelial cells, is of paramount importance for intestinal health and plays a pivotal role in effectively resisting the invasion of pathogenic microorganisms. However, small intestinal epithelial cells in the jejunum and ileum are the primary target cells of PEDV infection. The virus can proliferate within these cells, causing severe damage to their normal function and resulting in significant atrophy of small intestinal villi, ultimately leading to fatal diarrhea or vomiting (4). Therefore, the impairment of the normal functions of small intestinal epithelial cells by PEDV is closely associated with its pathogenesis and pathogenic mechanism.

Small intestinal epithelial cells possess a substantial number of mitochondria, which confer advantageous effects on their nutrient absorption and barrier function. Mitochondria are a type of semi-autonomous organelles with intricate structures. Its own genome encodes 13 subunits of the respiratory chain complex involved in oxidative phosphorylation systems, as well as 22 tRNA and 2 rRNA molecules, which are extensively involved in cellular biological processes such as signal transduction, ATP synthesis, innate immune response, autophagy, and apoptosis (5). Disruption of mitochondrial function can therefore affect a variety of cellular physiological or pathological processes, resulting in the occurrence and development of diseases. For certain enteroviral diseases, mitochondrial damage in intestinal cells is an important event in their pathogenesis. For example, rotavirus infection, a prominent cause of viral gastroenteritis in infants worldwide, induces mitochondrial dysfunction in virus-infected cells and subsequently triggers Bax-induced apoptosis. This process leads to the absorption dysfunction of small intestinal epithelial cell and consequent osmotic diarrhea (6). Additionally, in Klein’s disease, mitochondrial dysfunction in the intestinal epithelium is a core mechanism leading to the occurrence of ileitis (7–9). Therefore, the mitochondrial damage in porcine intestinal epithelial cells may occur during PEDV infection and play a critical role in the pathogenesis of PEDV.

miRNAs, a class of 20–25 nucleotide-noncoding RNA molecules widely present in eukaryotic cells, exert regulatory control on gene expression levels by binding to the 3′-untranslated region (UTR) of target mRNAs to either inhibit post-transcriptional translation or promote mRNA degradation (10, 11). In addition, miRNAs can also directly target CDS-regulated gene expression in the translation region (12–14). Mature miRNAs were initially believed to be primarily accumulated in the cytoplasm and exerted their gene regulatory functions, but mounting evidence suggests the presence of miRNAs in the mitochondria of eukaryotic cells (15–17). Furthermore, the expression profile of mitochondrial miRNAs differs from that in the cytoplasm, leading to their classification as mitochondrial miRNAs (mitomiRs). Accumulating studies have demonstrated that mitomiRs can modulate mitochondrial morphology, metabolism, redox homeostasis, autophagy, and apoptosis by regulating the expression of mitochondrion-related genes (18–20). However, the pathological mechanism underlying mitomiR-mediated regulation of mitochondrial damage in virus-infected cells remains to be investigated.

Based on existing literature and the important function of mitochondria in cellular energy metabolism and defense mechanisms, this study hypothesized that PEDV infection leads to mitochondrial dysfunction, mediated by specific miRNAs targeting mitochondrial genes. Thus, we first examined the specific mitochondrial damage induced by PEDV in porcine jejunum epithelial cells. Subsequent analyses revealed that PEDV infection stimulates miR-34c expression. We then delineated the influence and underlying mechanism of miR-34c on mitochondrial function and mitophagy in these cells. Additionally, we confirmed that CREB3L1 is a transcription factor that regulates miR-34c upstream. In summary, this study demonstrates for the first time that PEDV infection upregulates CREB3L1, which in turn regulates the expression of miR-34c and targets the mitochondrial gene COX1, thereby modulating mitochondrial damage in jejunum epithelial cells.

## RESULTS

### PEDV infection caused intestinal damage and mitochondrial disorder in jejunum epithelial cells *in vivo*

**Fig 1:**
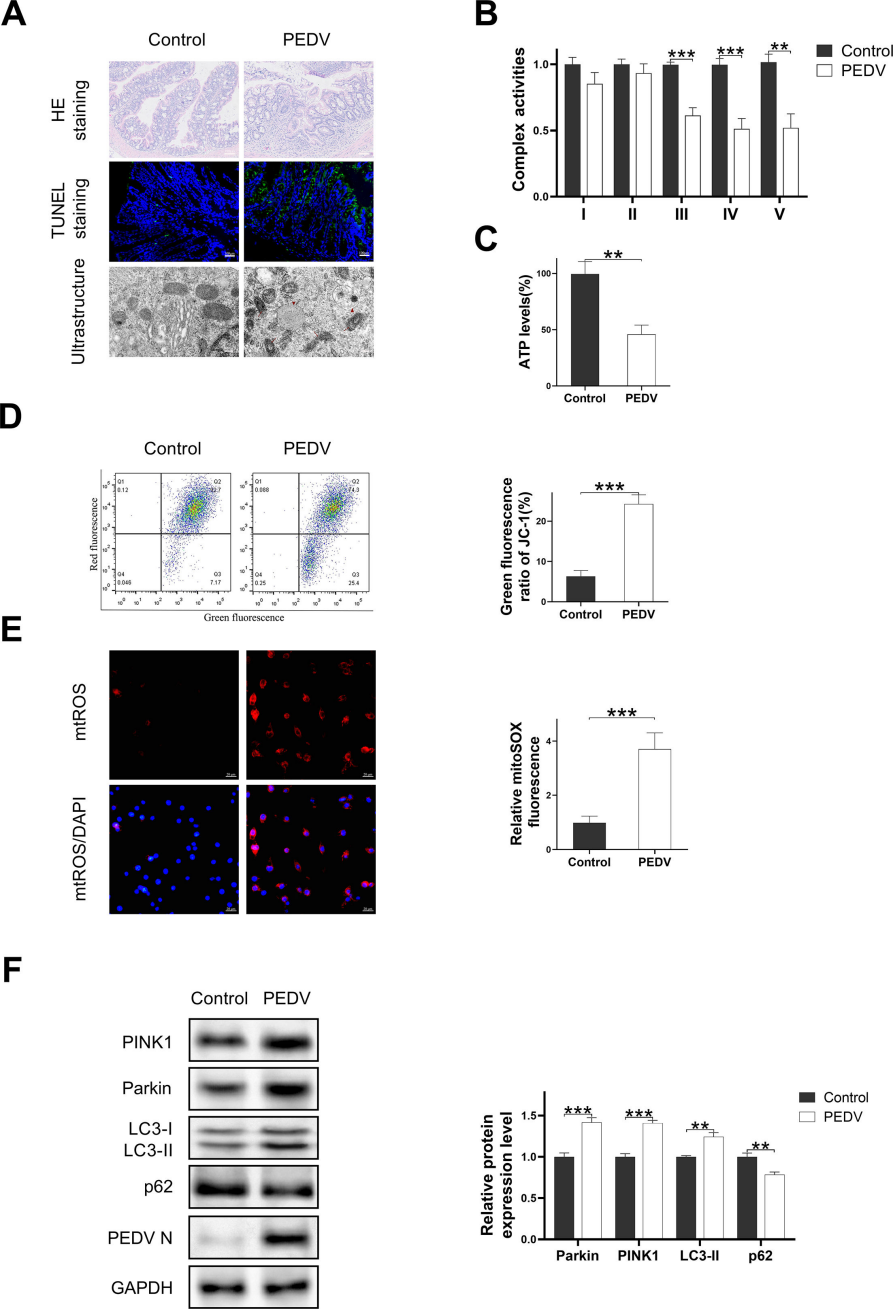
PEDV infection led to intestinal damage and mitochondrial disorder in small intestine epithelium. (**A**) HE staining, TUNEL staining, and ultrastructure in small intestine epithelium. Damaged mitochondria and mitophagosomes are indicated by red arrows and red triangles, respectively. Mitochondrial complex I–V activities (B) and the production of ATP (C) in small intestine epithelium at 3 days post-challenge. (**D**) The detection of MMP using through JC-1 probe. (**E**) Representative images of jejunum epithelial cells and the detection of mitochondrial reactive oxygen species (mtROS) using MitoSOX Red. (**F**) Western blot analysis for the expression levels of mitophagy-related protein (Parkin, PINK1, LC3-I, LC3-II, and p62) in small intestine epithelium at 3 days post-challenge. Data are presented as mean ± SD. (**P < 0.001 and ***P < ).0.0001

To explore the damage to the epithelium of porcine small intestine caused by PEDV infection and its impact on the mitochondrial function, a model of jejunum epithelial cells infected by PEDV *in vivo* was established using newborn piglets (Fig. S1A and B). The challenge group of piglets exhibited atrophic and shortened villi in the jejunum, with occasional shedding of jejunum epithelial cells, as depicted in Fig. 1A. TUNEL staining was employed to assess the extent of apoptotic injury of jejunum epithelial cells in piglets. Compared with that in the control group, a higher degree of positive cell staining was exhibited in the jejunal epithelium in the challenge group (Fig. 1A). The images of transmission electron microscopy (TEM) revealed disordered mitochondrial morphology, absence of mitochondrial cristae, and prominent formation of mitophagosomes in the jejunum epithelial cells in the challenge group, whereas these changes were not observed in the control group (Fig. 1A).

Subsequently, the impact of PEDV infection on the mitochondrial complex activity, ATP synthesis, mitochondrial membrane potential, mitochondrial ROS levels, and mitophagy-related protein expression was assessed in porcine jejunum epithelial cells under *in vivo* conditions. As depicted in Fig. 1B, the activities of mitochondrial complexes III, IV, and V were significantly reduced in jejunum epithelial cells in the challenge group, as compared with those in the control group (Fig. 1B), leading to a corresponding decrease in ATP production (Fig. 1C). Mitochondrial membrane potential is a crucial indicator for assessing mitochondrial damage, as it plays a vital role in maintaining the electron transport chain and ATP production (21). Analysis of jejunum epithelial cells isolated from pigs treated with the JC-1 probe, as determined by flow cytometry, revealed an increase in the proportion of cells with green fluorescence in the challenge group (Fig. 1D). The results indicate that PEDV infection leads to the reduction in mitochondrial membrane potential. On the other hand, mitochondrial ROS primarily originates from oxidative phosphorylation processes occurring within mitochondria (22), and the disruption of mitochondrial complex activity results in damage to the electron transport chain, leading to excessive production of mitochondrial ROS, a crucial event in the process of mitochondrial impairment (23). The fluorescence imaging results of jejunum epithelial cells treated by MitoSOX Red revealed a significant increase in red fluorescence intensity in the challenge group (Fig. 1E). These results indicate that PEDV infection induces the mitochondrial dysfunction in jejunum epithelial cells of piglets.

As the depolarization of the mitochondrial membrane potential and the increase in mitochondrial ROS levels are closely associated with the occurrence of mitophagy, the relationship between PEDV infection and mitophagy was then investigated. We assessed the expression levels of mitophagy-related proteins in jejunum epithelial cells by western blot analysis, revealing that PEDV infection induced upregulation of Parkin, PINK1, and LC3-II protein expression but reduced the p62 protein expression (Fig. 1F). The findings suggest that PEDV infection triggers the activation of the PINK1-Parkin pathway, leading to mitophagy. Furthermore, as PEDV infection induces the formation of mitophagy structures in jejunum epithelial cells (Fig. 1A), these results together provide evidence for the induction of mitophagy *in vivo* infection of PEDV.

### Mitochondrial miRNA and mRNA expression profiles were characterized and analyzed jointly

**Fig 2:**
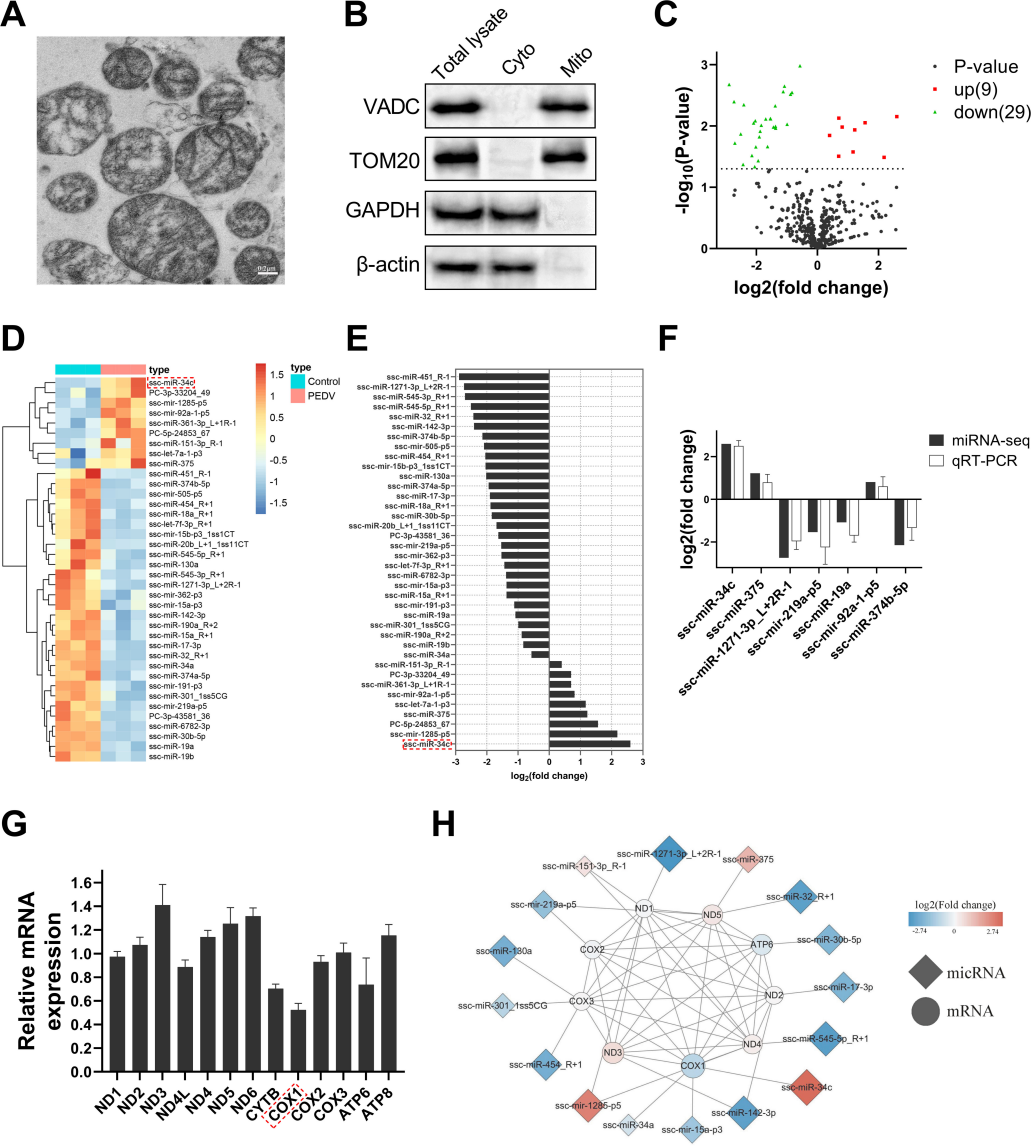
Characterization and integrated analysis of mitochondrial miRNA and mRNA expression profile. (**A**) TEM image of isolated mitochondria. (**B**) The purity of mitochondria was determined at the protein level by western blotting (VADC and TOM20 for mitochondria, GAPDH, and β-actin for cytosol). (**C**) Volcano plot showing the expression profile of differentially expressed mitomiRs in small intestine epithelium. The pink and blue plots represent upregulation and downregulation, respectively. (**D**) Heat map of mitomiRs in small intestine epithelium. (**E**) Histogram of differentially expressed mitomiRs in small intestine epithelium. (**F**) Verification of miRNA-seq results by qRT-PCR. (**G**) qRT-PCR detection of mitochondrial gene expression. (**H**) A miRNA-mRNA and their protein-protein interaction network of differentially expressed mitomiRs and mitochondrial genes. The size of diamonds and circles is proportional to the fold change of miRNA and mRNA. Data are presented as mean ± SD.

The regulation of gene expression by miRNA and the role of mitomiR as a crucial regulator in the mitochondrial damage have been widely acknowledged. In this study, we conducted small RNA sequencing on isolated and purified mitochondria from jejunum epithelial cells to identify potential mitomiRs involved in the mitochondrial dysfunction and intestinal injury during PEDV infection. The structural integrity and purity of the extracted and purified mitochondria were confirmed using TEM and western blot experiments (Fig. 2A and B). Subsequently, the analysis of small RNA sequencing results revealed 9 upregulated and 29 downregulated mitomiRs, with miR-34c exhibiting the most significant upregulation, while ssc-miR-451_R-1 displayed the most pronounced downregulation (Fig. 2C through E) (Table S2). Seven differentially expressed mitomiRs were further validated by quantitative real-time PCR (qRT-PCR), and the results demonstrated a consistent trend with the miRNA-seq data (Fig. 2F), thus confirming the reliability of the miRNA-seq analysis.

Considering the regulatory functions of miRNA in gene expression, it is postulated that differentially expressed mitomiR may exert an impact on the mitochondrial gene expression. Consequently, qRT-PCR was employed to measure the expression levels of 13 protein-coding genes in the mitochondrial genome of jejunum epithelial cells during PEDV infection. The data indicated that ND3, ND6, and ND5 genes exhibited significant upregulation, whereas COX1, CYTB, and ATP6 genes displayed significant downregulation (Fig. 2G). Subsequently, the targeting relationship between differentially expressed mitomiRs and protein-coding genes of mitochondrial genome was predicted using miRanda software (V3.3a) (Table S3), and the interaction between corresponding mitochondrial genome-coding proteins was analyzed through STRING v11.0 online statistical analysis software. The corresponding interaction network was constructed using Cytoscape v3.8.0 software (Fig. 2H). It was observed that miR-34c potentially targets COX1 and the significant upregulation of miR-34c expression appears to be strongly associated with the downregulation of COX1 expression. These findings suggest that PEDV may employ a regulatory mechanism involving miR-34c to modulate COX1 expression during infection.

### miR-34c targeted directly to COX1 and regulated its expression in PEDV-infected IPEC-J2 cells

**Fig 3:**
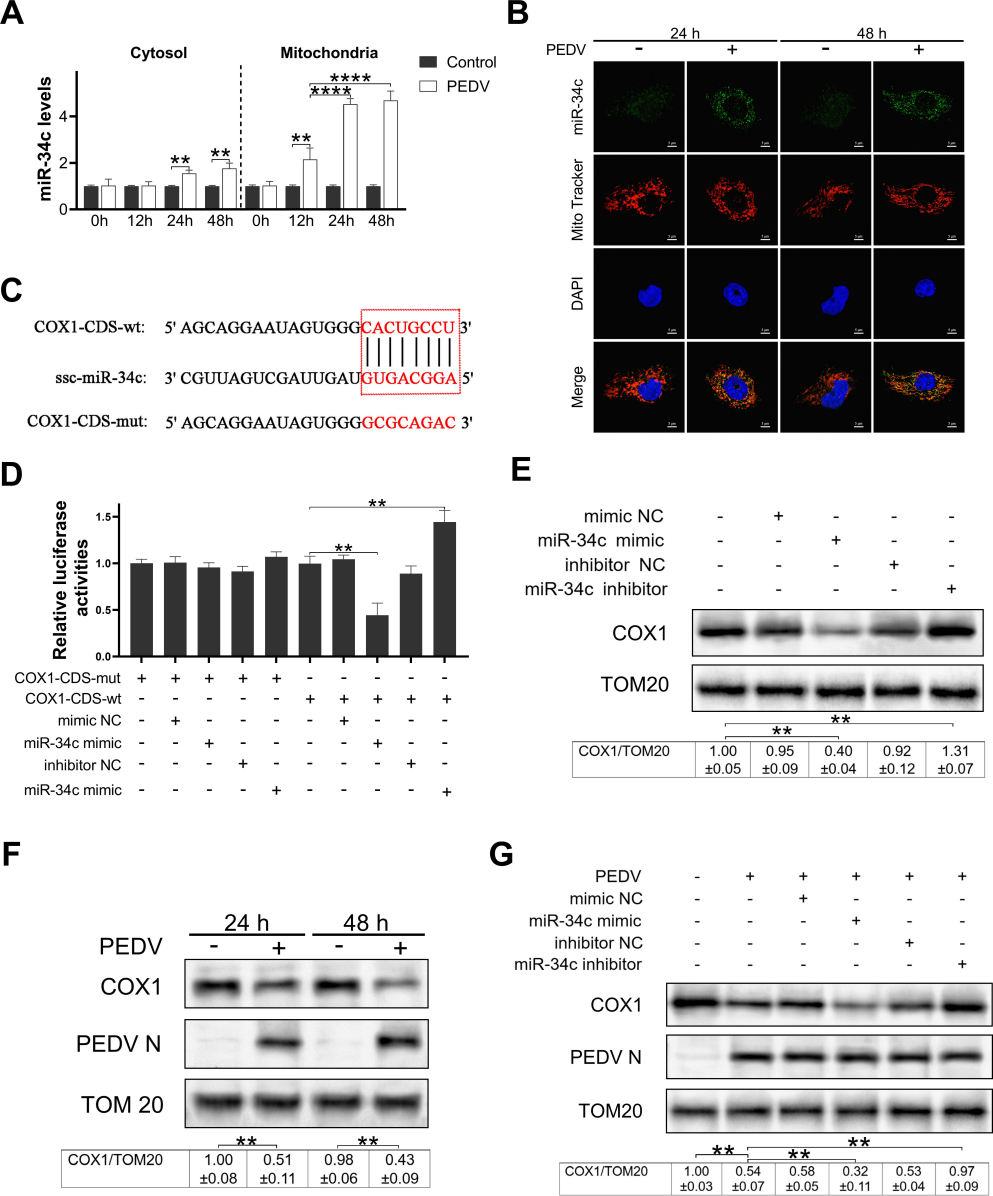
miR-34c targeted directly COX1 and regulated its expression in IPEC-J2 during PEDV infection. (**A**) The expression of miR-34c in the cytosol and mitochondria of IPEC-J2 cells infected with PEDV in a time-course experiment. (**B**) The localization and expression of miR-34c were determined by fluorescence *in situ* hybridization. (**C**) Analysis of the binding site in the COX1 coding region for miR-34c and the information on the mutation sites. (**D**) miR-34c significantly inhibited the translation of the luciferase gene by binding directly to the coding region of COX1. (**E**) Western blot results demonstrated the impact of miR-34c on COX1 expression. (**F**) The protein expression of COX1 during PEDV infection. (**G**) IPEC-J2 cells were infected with PEDV (multiplicity of infection = 0.01), following the transfection with mimic NC, miR-34c mimic, inhibitor NC, and miR-34c inhibitor alone or in combination, and the expression of COX1 was analyzed by western blotting. Data are presented as mean ± SD. (**P < 0.001 and ****P < 0.00001).

To further investigate the regulatory mechanism of mitomiR during PEDV infection, we established a model of IPEC-J2 cells infected by PEDV *in vitro*. Data showed that PEDV effectively infected IPEC-J2 cells, and the infection was significantly better at 48 hours post infection compared with 24 hours post infection (Fig. S2A and B). To ensure effective viral infection in each subsequent experiment, the PEDV genome copies or PEDV N protein levels in the cell culture supernatants of the control group and experimental group were parallelly detected (some data not shown). The expression of miR-34c in cytosolic and mitochondrial fractions was assessed using qRT-PCR at 0, 12, 24, and 48 hours post-PEDV infection (Fig. 3A). The expression of miR-34c in mitochondria was significantly upregulated with the progression of PEDV infection. FISH staining demonstrated that the fluorescence signal of miR-34c was markedly enhanced at 24 and 48 hours post infection and exhibited strong co-localization with mitochondria (Fig. 3B). These findings suggest that PEDV infection induces both the expression and translocation of miR-34c into mitochondria.

The regulatory role of miR-34c on the expression of COX1 was subsequently revealed by the presence of a potential binding site between miR-34c and COX1 (Fig. 3C). Luciferase reporter constructs containing wild-type or mutant COX1 were generated. The luciferase assay showed that either overexpression or knockdown of miR-34c did not have a distinct effect on the luciferase activity of COX1-mut (Fig. 3D). However, overexpression of miR-34c inhibits the translation of luciferase reporter gene containing wild-type COX1. Conversely, knockdown of miR-34c had an opposite effect, indicating that miR-34c directly binds to COX1.

Subsequently, we detected the expression of COX1 protein in IPEC-J2 cells. As anticipated, transfection with the miR-34c mimic resulted in the suppression of COX1 expression, whereas the miR-34c inhibitor promoted COX1 expression (Fig. 3E). In PEDV-infected cells, the expression of COX1 protein was significantly downregulated (Fig. 3F). Overexpression of miR-34c further enhanced the PEDV infection-induced suppression of COX1 protein expression, while knockdown of miR-34c reversed this inhibitory effect (Fig. 3G). These findings suggest that PEDV infection downregulates COX1 protein expression by inducing upregulation of miR-34c.

### PEDV infection induced the mitochondrial dysfunction and mitophagy in IPEC-J2 cells *in vitro*

**Fig 4:**
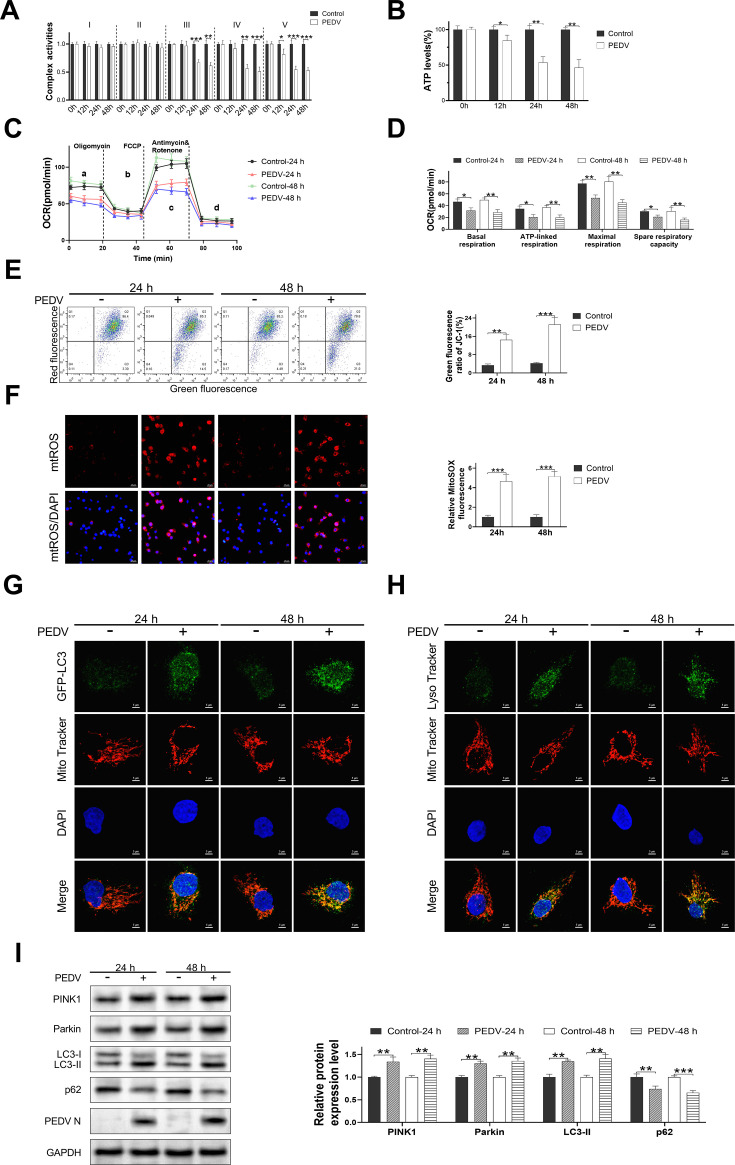
PEDV infection induced mitochondrial dysfunction and mitophagy in IPEC-J2 cells *in vitro*. IPEC-J2 cells were infected with PEDV (MOI = 0.01) for different times and mitochondrial complex I–V activities (A), ATP production (B), mitochondrial oxygen consumption (**C, D**), mitochondrial membrane potential (E), and mtROS levels in IPEC-J2 cells were detected. (**F**) Representative images of IPEC-J2 cells and the detection of mtROS using MitoSOX Red. Representative images of immunofluorescence double-labeling GFP-LC3 and MitoTracker (G), LysoTracker, and MitoTracker (H). (**I**) Western blot analysis of the protein changes related to mitophagy. Data are presented as mean ± SD. (*P < 0.05, **P < 0.001, and ***P < 0.0001).

Subsequently, we examined the impact of PEDV infection on the mitochondrial function and mitophagy in jejunal epithelial cells during *in vitro* infection. To achieve this, IPEC-J2 cell mitochondrial function was assessed at different times post-PEDV infection. The activity of mitochondrial complex III–V and ATP production exhibited a significant decline over the course of PEDV infection, particularly at 24 and 48 hours post infection (Fig. 4A and B). The reduction of mitochondrial complex III–V activities and the disturbance of ATP production may be accompanied by the alteration of the oxidative phosphorylation process. There was a significant decrease in the mitochondrial oxygen consumption rate, including the oxygen consumption rate (OCR) of basal respiration, ATP-linked respiration, maximal respiration, and spare respiratory capacity at 24 and 48 hours post infection (Fig. 4C and D). A notable diminution in the mitochondrial membrane potential and a substantial upregulation of mitochondrial ROS were observed during this period (Fig. 4E and F).

Moreover, co-transfection of GFP-LC3 and LysoTracker with MitoTracker was performed in IPEC-J2 cells to assess the mitophagy activity during PEDV infection. The results indicated a remarkable enhancement in the co-localization of GFP-LC3 and LysoTracker with MitoTracker at 24 and 48 hours post-PEDV infection, compared with the control group (Fig. 4G and H). Additionally, the protein expression levels of Parkin, PINK1, and LC3-II were significantly upregulated, while p62 was downregulated over time (Fig. 4I). These findings validated that the mitochondrial dysfunction and mitophagy were triggered by PEDV infection of IPEC-J2 cells *in vitro*.

### PEDV infection regulated the mitochondrial dysfunction and mitophagy via the miR-34c/COX1 axis

**Fig 5:**
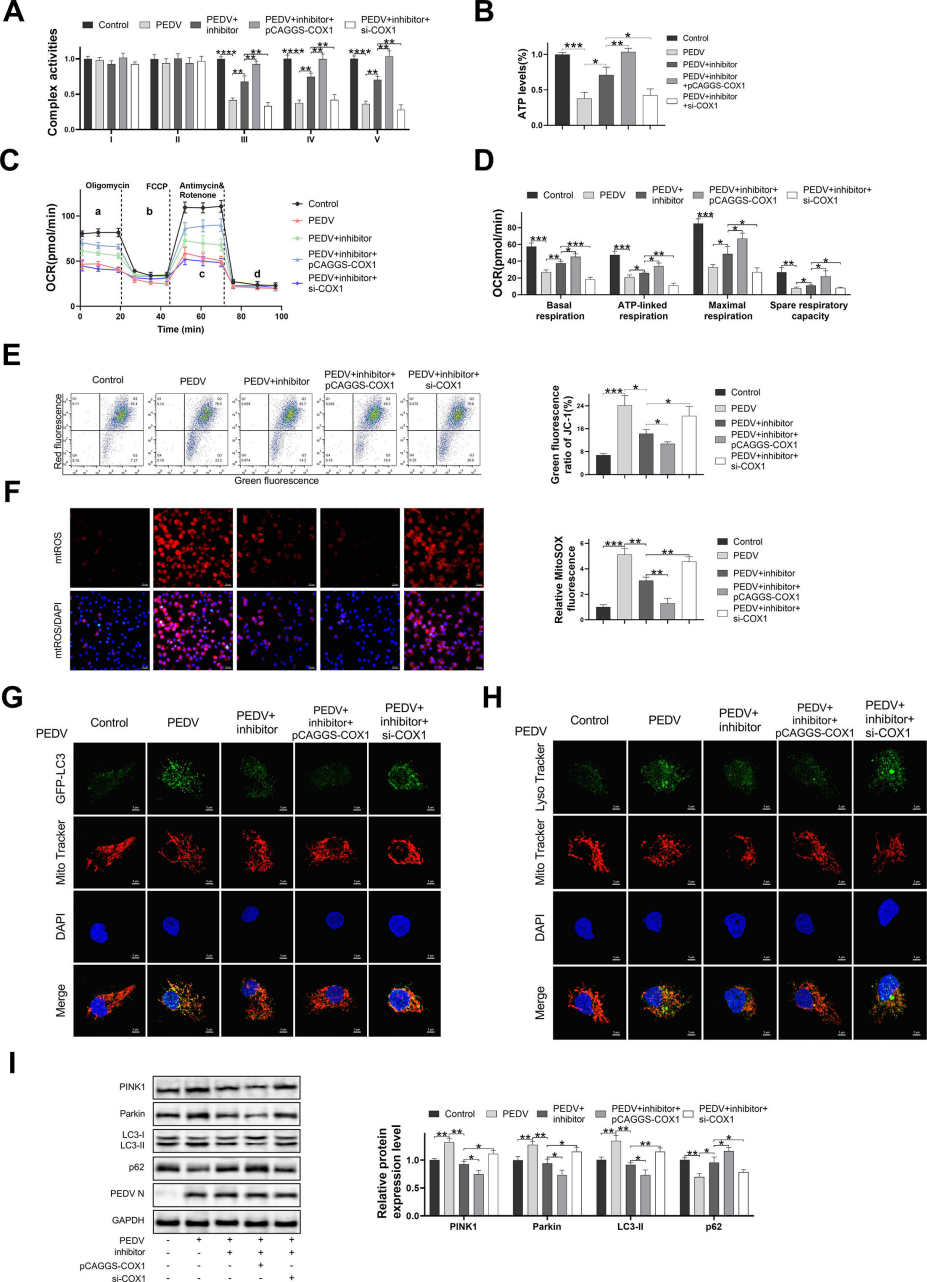
PEDV infection regulated mitochondrial dysfunction and mitophagy via the miR-34c/COX1 axis. IPEC-J2 cells were transfected with miR-34c inhibitor, pCAGGS-COX1, or si-COX1 alone or in combination and infected with PEDV (MOI = 0.01). After 48 hours, cells or culture superserum were collected for detection. (**A**) Mitochondrial complex I–V activities. (**B**) ATP production. (**C, D**) Mitochondrial oxygen consumption. (**E**) Mitochondrial membrane potential. (**F**) Representative images of IPEC-J2 cells and the detection of mtROS using MitoSOX Red. Representative images of immunofluorescence double-labeling GFP-LC3 and MitoTracker in IPEC-J2 cells (**G**), LysoTracker, and MitoTracker (H). (**I**) Expression levels of proteins related to mitophagy. Data are presented as mean ± SD. (*P < 0.05, **P < 0.001, ***P < 0.0001, and ****P < 0.00001).

The results of mitochondrial protein expressions in PEDV-infected IPEC-J2 cells exhibited that the COX1 was significantly downregulated (Fig. S3). To further investigate the involvement of the miR-34c/COX1 pathway in regulating the mitochondrial function and mitophagy during PEDV infection, pCAGGS-COX1 and si-COX1 were co-transfected with the miR-34c inhibitor into IPEC-J2 cells, respectively. Consequently, inhibition and overexpression models of miR-34c/COX1 were successfully established (Fig. S4A and B). The miR-34c inhibitor effectively mitigated the PEDV infection-induced mitochondrial dysfunction and mitophagy, and overexpression of COX1 significantly enhanced ATP production, mitochondrial complex III–V activities, mitochondrial oxygen consumption at different stages, and mitochondrial membrane potential, while reducing mitochondrial reactive oxygen species (mtROS) levels (Fig. 5A through F). Transfection of si-COX1 significantly attenuated ATP production, mitochondrial complex III–V activities, mitochondrial oxygen consumption at different stages, mitochondrial membrane potential, and augmented mtROS levels (Fig. 5A through F). Moreover, the fluorescence images obtained from laser confocal microscopy demonstrated that COX1 overexpression markedly diminished the colocalization rate of GFP-LC3 and LysoTracker with MitoTracker in the PEDV + miR-34c inhibitor treatment group (Fig. 5G and H). Knockdown of COX1 exerted an opposite effect (Fig. 5G and H). In comparison to PEDV + miR-34c inhibitor treatment, the addition of pCAGGS-COX1 in PEDV + miR-34c inhibitor + pCAGGS-COX1 treatment significantly attenuated the protein expression of Parkin, PINK1, and LC3-II and concurrently elevated the protein expression of p62 (Fig. 5I). Conversely, the group treated with PEDV + miR-34c inhibitor + si-COX1 exhibited contrasting outcomes (Fig. 5I). These findings suggest that the miR-34c/COX1 axis mediates the mitochondrial dysfunction and mitophagy during PEDV infection.

### CREB3L1 binds to the miR-34c promoter to activate the miR-34c/COX1 axis during PEDV infection

**Fig 6:**
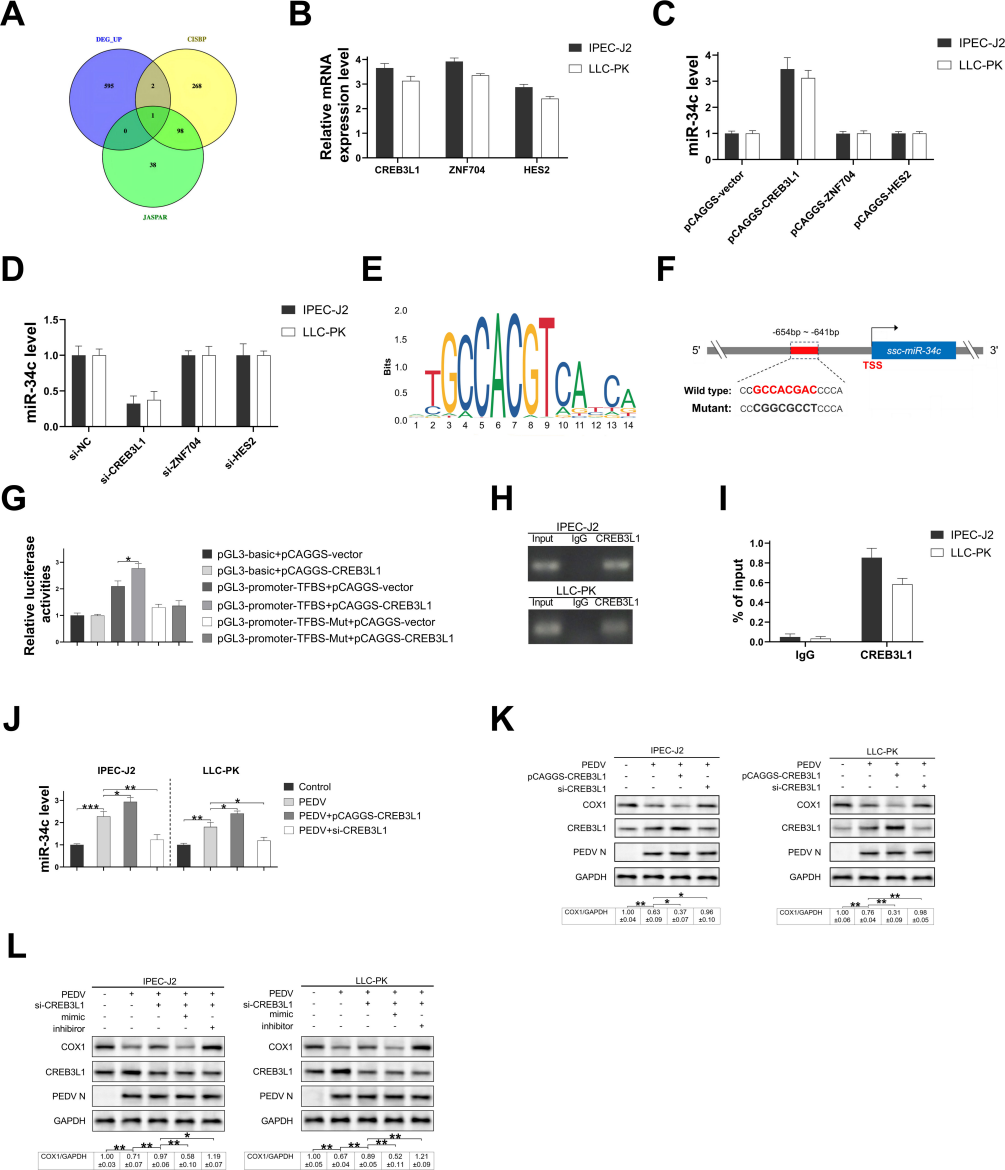
CREB3L1 binds to the miR-34c promoter to activate miR-34c/COX1 axis during PEDV infection. (**A**) Venn diagram analyzing the upregulated genes and predicted transcription factors from the JASPAR and CIS-BP database. (**B**) The expression of CREB3L1, ZNF704, and HES2 in IPEC-J2 and LLC-PK cells infected with PEDV (MOI = 0.01) for 48 hours. Transcription factors CREB3L1, ZNF704, and HES2 were overexpressed (C) or silenced (D) in IPEC-J2 and LLC-PK cells, and the expression of miR-34c was determined. (**E**) The binding motifs of CREB3L1 on the promoters of miR-34c. (**F**) Schematic diagram of the putative binding sites of CREB3L1 on the miR-34c promoter. (**G**) The miR-34c promoter sequence and mutated sequence were cloned to the upstream of the pGL3 vector and then co-transfected into 293T cells with pCAGGS-CREB3L1 or control vector. Luciferase activity was measured in each group of cells. The chromatin immunoprecipitation (ChIP) assay was performed in IPEC-J2 and LLC-PK cells transfected with pCAGGS-CREB3L1. Electrophoretic gel images (**H**) and ChIP-qPCR (I) were used to calculate the binding rates of the CREB3L1 and miR-34c promoters. (**J–K**) IPEC-J2 and LLC-PK cells were treated with pCAGGS-CREB3L1 and si-CREB3L1, respectively, and were infected with PEDV (MOI = 0.01) for 48 hours. Cells were collected to detect the mRNA levels of miR-34c (J) and the protein levels of COX1, CREB3L1, PEDV N, and GAPDH (K). (**L**) IPEC-J2 and LLC-PK cells were transfected with si-CREB3L1, miR-34c mimic, and miR-34c inhibitor alone or in combination and were infected with PEDV (MOI = 0.01) for 48 hours. Cells were collected to detect the protein levels of COX1, CREB3L1, PEDV N, and GAPDH. Data are presented as mean ± SD. (*P < 0.05, **P < 0.001, and ***P < 0.0001).

We investigated the upstream regulatory mechanisms of miR-34c upregulation during PEDV infection. Previous studies reported that PEDV infection of IPEC-J2 cells led to the upregulation of 598 genes (24). Prediction of potential transcription factors binding to the upstream region of the miR-34c promoter using the JASPAR and CIS-BP databases and a combined data analysis identified three potential transcriptional regulators CREB3L1, ZNF704, and HES2 (Fig. 6A). qRT-PCR analysis confirmed that PEDV infection upregulated the expression of CREB3L1, ZNF704, and HES2 in both IPEC-J2 and LLC-PK cells (Fig. 6B) and also increased mitochondrial miR-34c expression in LLC-PK cells (Fig. S5). Overexpression or knockdown of CREB3L1, ZNF704, and HES2 in these cell lines demonstrated that only CREB3L1 modulated miR-34c expression levels (Fig. S6A and B) (Fig. 6C and D). A binding motif for CREB3L1 was identified within the −654 to −641 bp region of the miR-34c promoter (Fig. 6E and F).

To verify the binding of CREB3L1 to the miR-34c promoter, luciferase reporter plasmids containing either wild-type or mutated miR-34c promoter sequences were constructed. Luciferase assays showed that CREB3L1 activated the wild-type miR-34c promoter but had no effect on the mutated promoter (Fig. 6G). Chromatin immunoprecipitation assays using CREB3L1-specific antibodies and qPCR primers targeting the miR-34c promoter sequence further confirmed the direct binding of CREB3L1 to the miR-34c promoter (Fig. 6H and I). Subsequent overexpression of CREB3L1 in PEDV-infected IPEC-J2 and LLC-PK cells significantly enhanced miR-34c expression and reduced COX1 expression, while CREB3L1 knockdown led to decreased miR-34c expression and increased COX1 expression (Fig. 6J and K). In addition, transfection of miR-34c mimic decreased the expression of COX1 in CREB3L1-knockdown IPEC-J2 and LLC-PK cells infected with PEDV, while transfection of the miR-34c inhibitor increased the expression of COX1 (Fig. 6L). These findings demonstrate that PEDV infection regulates the miR-34c/COX1 pathway through upregulation of CREB3L1.

### PEDV-induced CREB3L1 promotes mitochondrial dysfunction and mitophagy by modulating the miR-34c/COX1 axis

**Fig 7:**
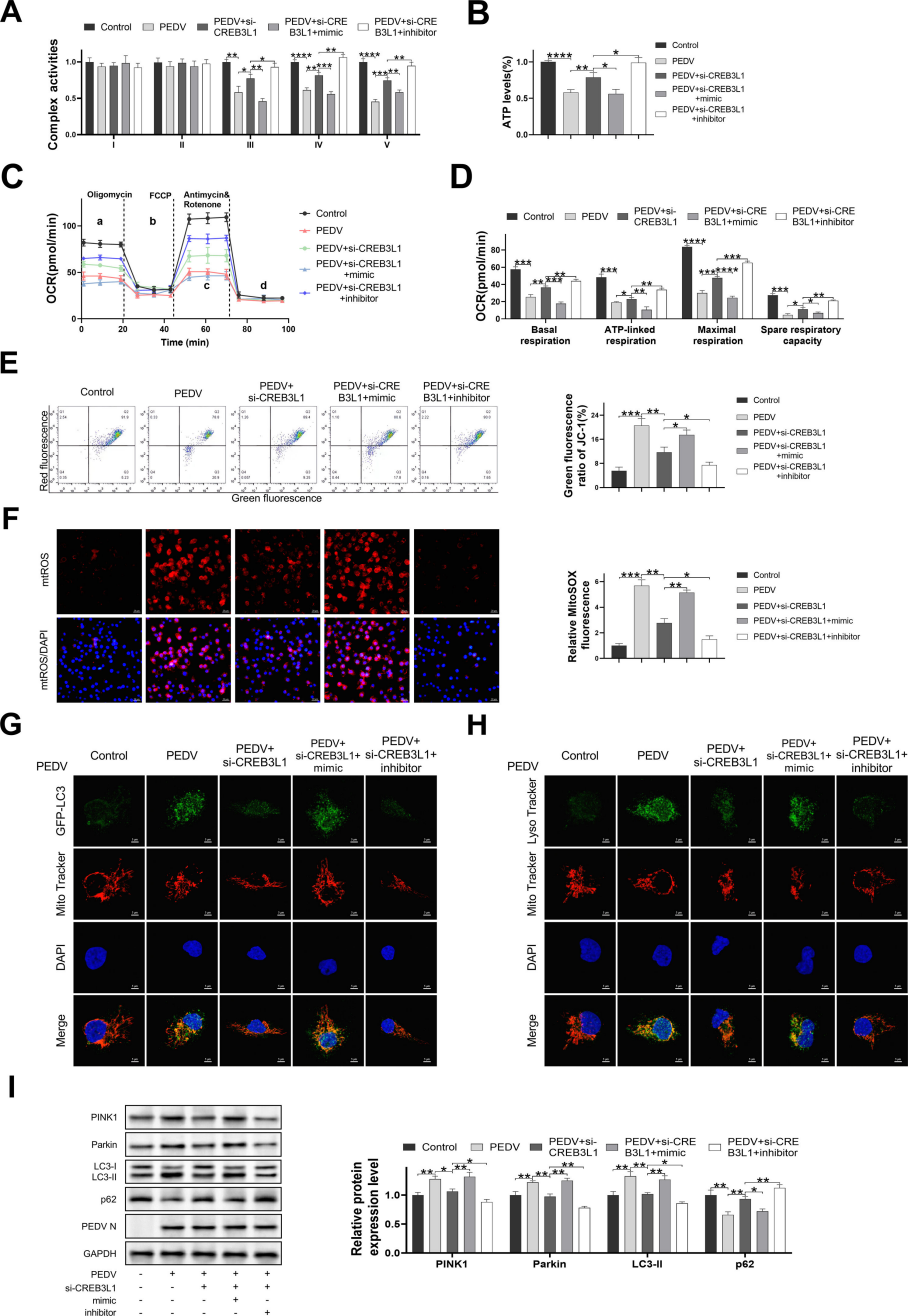
PEDV-induced CREB3L1 promotes mitochondrial dysfunction and mitophagy by modulating miR-34c/COX1 axis. IPEC-J2 cells were transfected with si-CREB3L1, miR-34c mimic, or miR-34c inhibitor alone or in combination and infected with PEDV (MOI = 0.01). After 48 hours, cells or culture superserum were collected for detection. (**A**) Mitochondrial complex I–V activities. (**B**) ATP production. (**C, D**) Mitochondrial oxygen consumption. (**E**) Mitochondrial membrane potential. (**F**) mtROS levels. Representative images of immunofluorescence double-labeling GFP-LC3 and Mito tracker in IPEC-J2 cells (**G**), LysoTracker, and MitoTracker (H). (**I**) Expression levels of proteins related to mitophagy. Data are presented as mean ± SD. (*P < 0.05, **P < 0.001, ***P < 0.0001, and ****P < 0.00001).

To determine whether CREB3L1 regulates the mitochondrial function and mitophagy via the activation of the miR-34c/COX1 axis during PEDV infection, IPEC-J2 cells were transfected, individually or in combination, with si-CREB3L1, miR-34c mimic, and miR-34c inhibitor. It was observed that si-CREB3L1 alleviated mitochondrial dysfunction and mitophagy induced by PEDV infection (Fig. 7A through F). Furthermore, transfection with the miR-34c mimic significantly decreased the activities of mitochondrial complexes III, IV, and V; ATP production, mitochondrial oxygen consumption; and mitochondrial membrane potential, while increasing mtROS levels (Fig. 7A through F). In addition to the direct observation that miR-34c significantly reduced the activity of mitochondrial complex IV (Cox1 is a component of respiratory chain complex IV), we also observed that complexes III and V decreased significantly. Previous studies suggested that the ATPase product ATP inhibits the activity of IV, suggesting that they may influence each other’s activity (25). In addition, the decrease in complex IV activity may reduce the proton pumping efficiency and reduce the proton gradient and potential difference across the membrane, thereby directly affecting the ATP synthesis capacity of complex V (ATP synthase). Therefore, we speculate that PEDV infection directly inhibits the activity of complex IV and then indirectly inhibits the activities of complexes III and V.

Conversely, transfection with the miR-34c inhibitor significantly increased the activities of mitochondrial complexes III, IV, and V; ATP production; mitochondrial oxygen consumption; and mitochondrial membrane potential and decreased mtROS levels (Fig. 7A through F). Additionally, transfection with the miR-34c mimic significantly enhanced the colocalization rate of GFP-LC3, LysoTracker, and MitoTracker in the PEDV + si-CREB3L1 treatment group, whereas the miR-34c inhibitor showed the opposite effect (Fig. 7G and H). Moreover, transfection with the miR-34c mimic notably restored the decreased expression levels of Parkin, PINK1, and LC3-II proteins induced by si-CREB3L1 and also reduced the expression levels of p62 protein (Fig. 7I). In contrast, transfection with the miR-34c inhibitor showed opposite results (Fig. 7I). These findings confirm that CREB3L1 upregulation by PEDV infection regulates mitochondrial dysfunction and mitophagy through the miR-34c/COX1 axis.

## DISCUSSION

PEDV is a prototypical enteropathogenic coronavirus that induces mortality in piglets. This virus primarily infects small intestinal epithelial cells via the digestive tract, resulting in villus atrophy and exfoliation within the small intestine as well as severe watery diarrhea and atrophic enteritis (4). By conducting experiments *in vitro* and *in vivo*, we have discovered that mitochondrial dysfunction plays a crucial role in the pathological process of PEDV infection in small intestinal epithelial cells, including notable changes in mitochondrial morphology, reduction of mitochondrial complex III–V activities, ATP production, mitochondrial oxygen consumption, and mitochondrial membrane potential, as well as accumulation of mitochondrial ROS. In addition, the expression of mitophagy-related proteins was significantly upregulated during PEDV infection. A strong colocalization between mitochondria and GFP-LC3 as well as mitochondria and lysosomes was observed in IPEC-J2 cells after 24 and 48 hours of PEDV infection *in vitro*, indicating the simultaneous induction of mitophagy by PEDV infection.

It is widely recognized that pathological alterations in the intestinal epithelium play a key role in the pathogenesis of PEDV infection. The abundance of mitochondria within the structural characteristics of intestinal epithelial cells underscores their physiological significance. The pathogenesis of ulcerative colitis, characterized by a significant reduction in mitochondrial complex II, III, and IV activities, is attributed to mitochondrial dysfunction leading to insufficient energy supply to the colonic epithelium (26, 27). The invasion of certain pathogens can also induce damage to the structure and function of mitochondria. The interplay between mitochondrial activity and intestinal barrier integrity during viral infections highlights a complex relationship where viral manipulation of host cellular mechanisms leads to significant physiological consequences. Inhibition of mitochondrial function could lead to intestinal barrier damage and immune compromise. T84 epithelial cells exposed to *Escherichia coli-LF82* exhibit mitochondrial swelling and fragmentation, depolarization of mitochondrial membrane potential, and reduction in ATP levels (28). Respiratory syncytial virus infection results in the fragmentation and redistribution of mitochondrial morphology within host cells, accompanied by impaired respiratory function, reduced membrane potential, and accumulation of ROS within mitochondria (29). During hepatitis B virus (HBV) infection, the HBV X protein is encoded and binds to the voltage-dependent anion channel (VDAC), leading to co-localization with mitochondria. This results in mitochondrial damage through regulation of mitochondrial membrane potential, Ca^+^ levels, and ROS levels (30–32). A recent study has demonstrated that severe acute respiratory syndrome coronavirus 2 (SARS-CoV-2) infection induces mitochondrial fission, impairs mitochondrial oxidative phosphorylation, reduces ATP levels, and disrupts ROS homeostasis in host bronchial epithelial cells, ultimately leading to apoptosis (33).

miRNAs have long been recognized as regulatory molecules controlling intracellular gene expression. Recent research has demonstrated that miRNAs can penetrate mitochondria and participate in the regulation of mitochondrial gene expression, thereby influencing the mitochondrial function and cellular processes. miR-1 has been observed to localize within mitochondria of mouse myoblasts (C2C12), where it forms a complex with AGO2 to directly target Cox1, Nd1, Cytb, Cox3, and Atp8 for enhanced protein synthesis and ATP production (20). The nuclear gene-encoded miR-181c can translocate to mitochondria and inhibit the expression of mt-COX1 protein, thereby impacting complex IV remodeling, levels of mitochondrial membrane potential, and production of ROS (18, 34). miR-2393 exerts inhibitory effects on the transcriptional levels of several mitochondrial genes, including ND2, ND4, ND5, CYTB, and COX1 by interacting with AGO2, and ultimately leads to alterations in mitochondrial metabolic reprogramming and apoptosis (35). miR-762 can bind to the coding sequence of ND2 and modulate its protein expression level, thereby impacting the activity of mitochondrial complex I and the production of ATP (19). Similarly, miR-1285 directly targets the 3′-UTR of ND2 and inhibits its expression, ultimately resulting in mitochondrial dysfunction and mitophagy in jejunal epithelial cells (36). Here, evaluation of the expression profile of mitochondrial miRNAs during PEDV infection discovered that PEDV infection induces the upregulation of miR-34c and its subsequent translocation to mitochondria to repress COX1 expression. The findings from dual-luciferase reporter assay, as well as the experiments *in vitro* involving miR-34c overexpression and knockdown, further corroborate the observation.

miR-34c plays diverse roles in various pig tissues and cells, exhibiting differential expression patterns during different stages of porcine testicular development. It targets platelet-derived growth factor receptor alpha, thereby suppressing its expression to modulating spermatogenesis and testicular morphogenesis (37, 38). Interestingly, miR-34c can interact with Notch1 in pluripotent stem cells (PSC) to establish a regulatory circuit governing their proliferation and differentiation processes (39). Furthermore, miR-34c regulates neuronal migration and cortical morphogenesis during porcine embryonic development by targeting DCX, as well as affecting cell proliferation ability and pluripotency through targeting c-Myc in piPSC-like cells (40, 41). Our previous research has demonstrated that miR-34c specifically targets COX1 and modulates its expression. COX1, a mitochondrial gene-encoded core subunit, is an integral component of complex IV in the respiratory chain (42). The respiratory chain complex IV facilitates the transfer of electrons from cytochrome c to oxygen molecules and the concomitant transport of protons across the membrane, thereby promoting ATP synthesis (43). It is evident that any impairment in COX1 function would directly impact mitochondrial oxidative phosphorylation (44). Previous research has demonstrated that certain miRNAs participate in the regulation of mitochondrial function and energy metabolism by targeting COX1 expression (18, 20, 34, 35). In this study, PEDV infection was shown to regulate the mitochondrial dysfunction and mitophagy in jejunal epithelial cells through the miR-34c/COX1 axis.

CREB3L1 is a bZIP transcription factor found in mammalian cells (45). Initially identified in astrocytes (46), the N-terminal of CREB3L1 dynamically regulates the expression of downstream Gcm1 by binding with the N-terminals of CREB4 and Luman, thus controlling astrocyte differentiation (47, 48). Research has shown that CREB3L1 is an important transcriptional regulator involved in neuroendocrine processes in the central nervous system. It acts on the CRE site and G-box element within the −170 to −120 bp region of the arginine vasopressin (AVP, a neurohypophysial hormone) promoter, activating AVP transcription (49). Luciferase assays and chromatin immunoprecipitation analyses have also demonstrated that CREB3L1 transcriptionally regulates by binding to the G-box motif in the proprotein convertase subtilisin/kexin type 1 promoter, thus participating in the secretion of proglucagon, vasopressin, and oxytocin in endocrine cells (50). Additionally, as a transcriptional regulator, CREB3L1 is found in bone, intestinal tissue, salivary glands, prostate, and various tumor cells, where it plays significant physiological and pathological regulatory roles (51–56). Here, through gene overexpression and knockdown experiments, luciferase reporter assays, chromatin immunoprecipitation, qRT-PCR, and western blotting, we demonstrated that PEDV infection upregulates the expression of the transcription factor CREB3L1, which binds to the miR-34c promoter region, thereby activating the miR-34c/COX1 axis. Our subsequent findings also indicate that CREB3L1, induced by PEDV infection, regulates mitochondrial dysfunction and mitophagy in jejunum epithelial cells by activating the miR-34c/COX1 axis. Intriguingly, Denard et al. (57) verified that CREB3L1 is a host factor that plays an important role in limiting virus spread by inhibiting the proliferation of virus-infected cells, such as murine γ-herpesvirus 68, HCV, West Nile virus, and Sendai virus. In our study, we further identified that PEDV infection activates the miR-34c/COX1 axis via the transcription factor CREB3L1 and the underlying biological processes were also explored. The specificity of the CREB3L1-miR-34c axis concerning PEDV infection is an area requiring further investigation; more studies should aim to elucidate whether this regulatory pathway exhibits unique characteristics or responses when faced with PEDV compared with other viruses.

**Fig 8:**
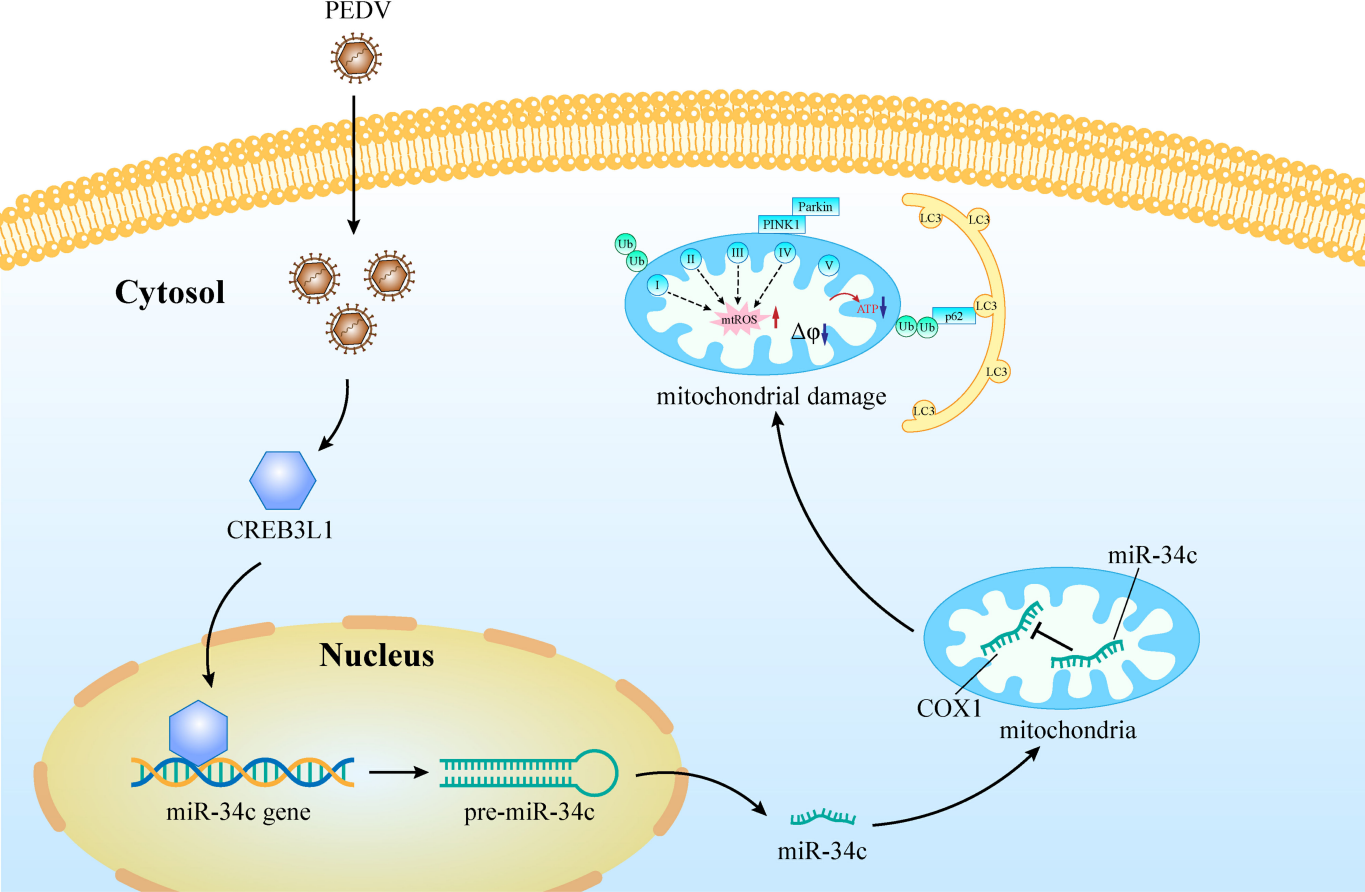
Schematic diagram of PEDV-induced mitochondrial damage through the CREB3L1/miR-34c/COX1 axis.

In summary, this study initially explores the regulation pattern of mitochondrial miRNAs in host cells during PEDV infection and finds that PEDV-induced nuclear miR-34c expression, translocated to mitochondria, inhibits COX1 translation, leading to mitochondrial dysfunction and mitophagy. Furthermore, PEDV infection upregulates the expression of the transcription factor CREB3L1, which activates the miR-34c/COX1 axis, thereby regulating mitochondrial damage in jejunum epithelial cells (Fig. 8). These results provide a new perspective on the regulation of mitomiRs and mitochondrial damage during PEDV infection, suggesting that mitomiRs might be novel targets against viral infections.

## MATERIALS AND METHODS

### Animal experiments

A total of 10 newborn piglets, free from PRRSV, PRV, and PEDV infection, were randomly allocated into two groups (five piglets/group). One group was subjected to a challenge with 2 mL dose of 10^6.5^ tissue culture infectious dose 50% (TCID_50_) of PEDV, while the other group received an equivalent volume of normal Vero cell culture supernatant as a negative control. Anal fecal swabs were collected from piglets prior to and at 12-hour intervals following the challenge, while the clinical manifestations of piglets were monitored. Three days post infection, piglets were humanely euthanized for sample collection.

### Cell culture and virus

IPEC-J2 cells were cultured in DMEM containing with 10% fetal bovine serum (10099141C, FBS, Gibco) in 5% CO_2_ at 37℃. The PEDV strain was previously isolated from a typical symptom of clinically infected swine and stored at our laboratory. IPEC-J2 cells were infected with PEDV (MOI = 0.01) in serum-free DMEM for 1 hour, after which the medium was replaced with serum-free DMEM containing 4 µg/mL trypsin (25200072, Gibco, USA) to support PEDV infection.

### Isolation of small intestinal epithelial cells and mitochondria

The abdominal cavities of pigs were dissected, and a section of jejunum about 20 cm long was cut out. Jejunum epithelial cells were isolated with the “enzyme + extra wash” method as previously described with modifications (58). In addition, density gradient centrifugation was used for mitochondria isolation of jejunum epithelial cells and IPEC-J2 cells as previously described (59).

### Histopathological and ultrastructural analyses

Formalin-fixed representative jejunum tissues were paraffin embedded and sectioned into 5 µm thickness, as previously described (60). Hematoxylin and eosin staining was performed on the sections. A TUNEL kit was used to process small bowel tissue sections according to the instructions. For ultrastructural analysis, samples were prepared following the procedure described by Wang et al. (61) and observed using TEM.

### ATP determination

ATP content in tissues and cells was determined by spectrophotometry using an ATP assay kit (Beyotime, China) according to the manufacturer’s protocol.

### Measurement of the mitochondrial OCR

According to the manufacturer’s protocol, the OCR of the cells was determined using the Extracellular Flux Analyzer (Seahorse XFe96, USA). Oligomycin (ATPase inhibitor), trifluoromethoxy carbonylcyanide phenylhydrazone (FCCP) (OXPHOS inhibitor), antimycin (complex III inhibitor) ,and rotenone (complex I inhibitor) were used to assess the OCR of basal respiration, ATP-linked respiration, maximal respiration, and spare respiratory capacity. As in Fig. 4C and 5C, a, b, c, and d represent the mitochondrial oxygen consumption rate in four different cell states (the OCR of basal respiration = a − d, the OCR of ATP-linked respiration = a − b, the OCR of maximal respiration = c − d, and the OCR of spare respiratory capacity = c − a).

### Determination of mitochondrial respiratory chain complex activities

Mitochondrial complex I/NADH-CoQ reductase Activity Assay Kit (BC0510), Mitochondrial complex II/succinate-coenzyme Q reductase Activity Assay Kit (BC3230), Mitochondrial complex III/CoQ-cytochrome C reductase Activity Assay Kit (BC3240), Mitochondrial Complex IV/Cytochrome C Oxidase Activity Assay Kit (BC0940), and Mitochondrial Complex V/ATP Synthase Activity Assay Kit (BC1440) were purchased from Beijing Solarbio Science & Technology Co. Ltd. And the activities of mitochondrial respiratory chain complexes I–V were measured by spectrophotometry according to the manufacturer’s instructions.

### Mitochondrial membrane potential assessment

A mitochondrial membrane potential assay kit with JC-1 (M8650, Solarbio, China) was used to detected mitochondrial membrane potential according to the manufacturer’s manual.

### mtROS measurements

According to the manufacturer’s instructions, 5 µM MitoSOX Red Mitochondrial Superoxide Indicator (40778ES50, Yeasen Biotechnology [Shanghai] Co. Ltd., China) was incubated with cells at 37°C for 10 minutes against light, and mtROS levels were measured by red fluorescence intensity using a fluorescence microscope. To ensure the accuracy of our mitochondrial superoxide measurement, we have included4',6-diamidino-2-phenylindole (DAPI) staining to delineate the cell nuclei. We further employed a method where the red fluorescence intensity is divided by the number of cells (as determined by DAPI staining) to quantify the mitoSOX-positive cell ratio.

### miRNA sequencing

Here, 1 ng of total miRNA was used to prepare miRNA sequencing libraries. The experimental procedure was performed according to the standard steps provided by Illumina, including library preparation and sequencing experiments. TruSeq Small RNA Sample Prep Kits (Illumina, San Diego, USA) were used for small RNA sequencing library preparation. After library preparation, the constructed libraries were sequenced using Illumina HiSeq 2000/2500 with a single-end 1 × 50 bp read length.

### Prediction of differentially expressed miRNA-mRNA interaction and protein-protein interaction

The targeting relationship between differentially expressed miRNAs and proteins coding genes in mitochondrial genome was predicted using the miRanda software (V3.3a) (Memorial Sloan-Kettering Cancer Center, New York, NY, USA) (62). The interactions between proteins encoded by mitochondrial genomes were analyzed by using STRING v11.0 online statistical analysis software (http://string-db.org/). Finally, those interactive relationships were visualized by using Cytoscape v3.8.0 software.

### Fluorescence *in situ* hybridization

Mitochondria were labeled with MitoTracker Red CMXRos (Yeasen, 40741ES50) following the manufacturer’s protocol in IPEC-J2 living cells. After fixation with 4% paraformaldehyde, FITC-labeled fluorescent probes targeting miR-34c were used for *in situ* hybridization and washed. Finally, the treated samples were observed under a confocal microscope (Leica, TCS-SP8).

### Cell transfection

The coding sequences of porcine COX1 and PINK1 were obtained and cloned into the PCAGGS vector. Additionally, siRNA targeting COX1 and PINK1, siNC, miR-34c mimic, mimic NC, miR-34c inhibitor, and inhibitor NC were purchased from Tsingke Biotechnology Co. Ltd. Cell transfection was performed using Lipofectamine 2000 (Invitrogen, 11668-019) following the manufacturer’s instructions. Subsequently, cells were collected for further assays.

### Luciferase reporter assays

The miR-34c mimic, mimic NC, miR-34c inhibitor, and inhibitor NC were co-transfected into 293T cells along with the pGL3 luciferase reporter vector containing wild-type or mutant COX1 sequences. At 48 hours after transfection, the cells were harvested, and luciferase activity was measured using the Dual Luciferase Reporter Gene Assay Kit (Beyotime, RG027).

### Visualization of mitochondria and GFP-LC3, mitochondria, and lysosome

For labeling the mitochondria and GFP-LC3, cells were initially infected with GFP-LC3 adenovirus (Hanbio, China) for 24 hours followed by incubation with MitoTracker Red CMXRos (Yeasen, 40741ES50) for 30 minutes. For visualizing the mitochondria and lysosomes, IPEC-J2 cells were stained at 37°C for 30 minutes using MitoTracker Red CMXRos (Yeasen, 40741ES50) and LysoTracker Green DND-26 (Yeasen, 40738ES50) according to the manufacturer’s protocol. Finally, fluorescence signals were visualized using a confocal microscope (Leica, TCS-SP8).

### Chromatin immunoprecipitation

A chromatin immunoprecipitation (ChIP) assay kit (Beyotime, P2078) was utilized following the specified protocol. Briefly, IPEC-J2 and LLC-PK cells transfected with pCAGGS-CREB3L1 were cross-linked using 1% formaldehyde, followed by cell collection and immunoprecipitation using CREB3L1 Antibody (Invitrogen, PA5-13537). Finally, the CHIP products were analyzed using qRT-PCR. The primers used for ChIP-qPCR are provided in Table S4.

### qRT-PCR analysis

The total RNA was extracted using TRIzol reagent (Solarbio, 15596026) and transcribed into cDNA using PrimeScript RT Master Mix (Takara, RR036A). Subsequently, qRT-PCR was performed using SYBR Premix Ex Taq II (Takara, RR820A) and specific primers (Table S1). The relative expression levels of the target genes were normalized by GAPDH.

### Western blot analysis

The western blot analysis was conducted according to the previously established protocol (63). Cell lysates were quantified for protein using the BCA Protein Assay Kit (Bio-Rad, 5000201) and subsequently subjected to SDS-PAGE before being transferred onto nitrocellulose membranes for subsequent western blot analysis.

### Statistical analysis

The data were presented as mean ± SD. Three biological replicates were prepared for each group (*n* = 3). Statistical analyses were conducted using GraphPad Prism Software (version 9.5, GraphPad Software Inc.). The significance of differences in the means was determined by a one-way analysis of variance method.

## Data Availability

The miRNA sequencing data have been submitted to the NCBI Sequence Read Archive (SRA) under accession numbers SRR25786758, SRR25786759, SRR25786760, SRR25786761, SRR25786762, and SRR25786763.

## SUPPLEMENTAL MATERIAL

Supplemental tables and figures jvi.00591-24-s0001.docx Tables S1 to S3; Fig. S1 to S6.

Table S4 jvi.00591-24-s0002.xlsx List of up-regulated genes and predicted transcription factors in the Venn diagram.

ASM does not own the copyrights to Supplemental Material that may be linked to, or accessed through, an article. The authors have granted ASM a non-exclusive, world-wide license to publish the Supplemental Material files. Please contact the corresponding author directly for reuse.
